# Mechanism of ferroptosis resistance in cancer cells

**DOI:** 10.20517/cdr.2024.127

**Published:** 2024-11-20

**Authors:** Yuan Wang, Guifang Yu, Xin Chen

**Affiliations:** ^1^Key Laboratory of Biological Targeting Diagnosis, Guangzhou Municipal and Guangdong Provincial Key Laboratory of Protein Modification and Disease, Therapy and Rehabilitation of Guangdong Higher Education Institutes, The Fifth Affiliated Hospital, Guangzhou Medical University, Guangzhou 511436, Guangdong, China.; ^2^Guangzhou Municipal and Guangdong Provincial Key Laboratory of Protein Modification and Disease, School of Basic Medical Sciences, Guangzhou Medical University, Guangzhou 511436, Guangdong, China.

**Keywords:** Ferroptosis resistance, lipid peroxidation, iron, cancer, antioxidant defenses

## Abstract

Ferroptosis is an iron-dependent cell death characterized by increased intracellular lipid peroxidation. Inducing ferroptosis has shown significant potential in eliminating various malignancies. However, the effectiveness of ferroptosis-based treatments is hampered by the intrinsic or acquired resistance of some tumors. In this review, we delineate the known mechanisms that regulate ferroptosis sensitivity and summarize the therapeutic application of ferroptosis inducers in cancer. Additionally, we discuss the roles of diverse signaling pathways that contribute to ferroptosis resistance in cancer cells, including the glutathione (GSH) and coenzyme Q (CoQ) pathways, NFE2-like bZIP transcription factor 2 (NRF2) antioxidant response, and lipid and iron metabolism. This emerging knowledge may serve as a foundation for developing novel anticancer strategies to overcome ferroptosis resistance.

## INTRODUCTION

Ferroptosis is a type of cell death distinct from apoptosis that depends on the presence of redox-active iron^[1]^. Unlike other forms of cell death, ferroptosis has unique features including accumulation of intracellular free iron, activation of pro-oxidative enzymes, altered mitochondrial morphology, and remodeling of polyunsaturated fatty acid (PUFA)-containing lipid^[2-4]^. These events lead to elevated levels of lipid peroxidation, which, when sufficiently accumulated, become lethal to cells. Consequently, ferroptosis is tightly controlled by iron metabolism, oxidative stress, and lipid metabolism^[5]^. This process plays a critical role in tumor suppression and can be triggered in various disease conditions^[6]^. Therefore, there is great interest in uncovering the regulatory mechanisms of ferroptosis.

**Figure 1:**
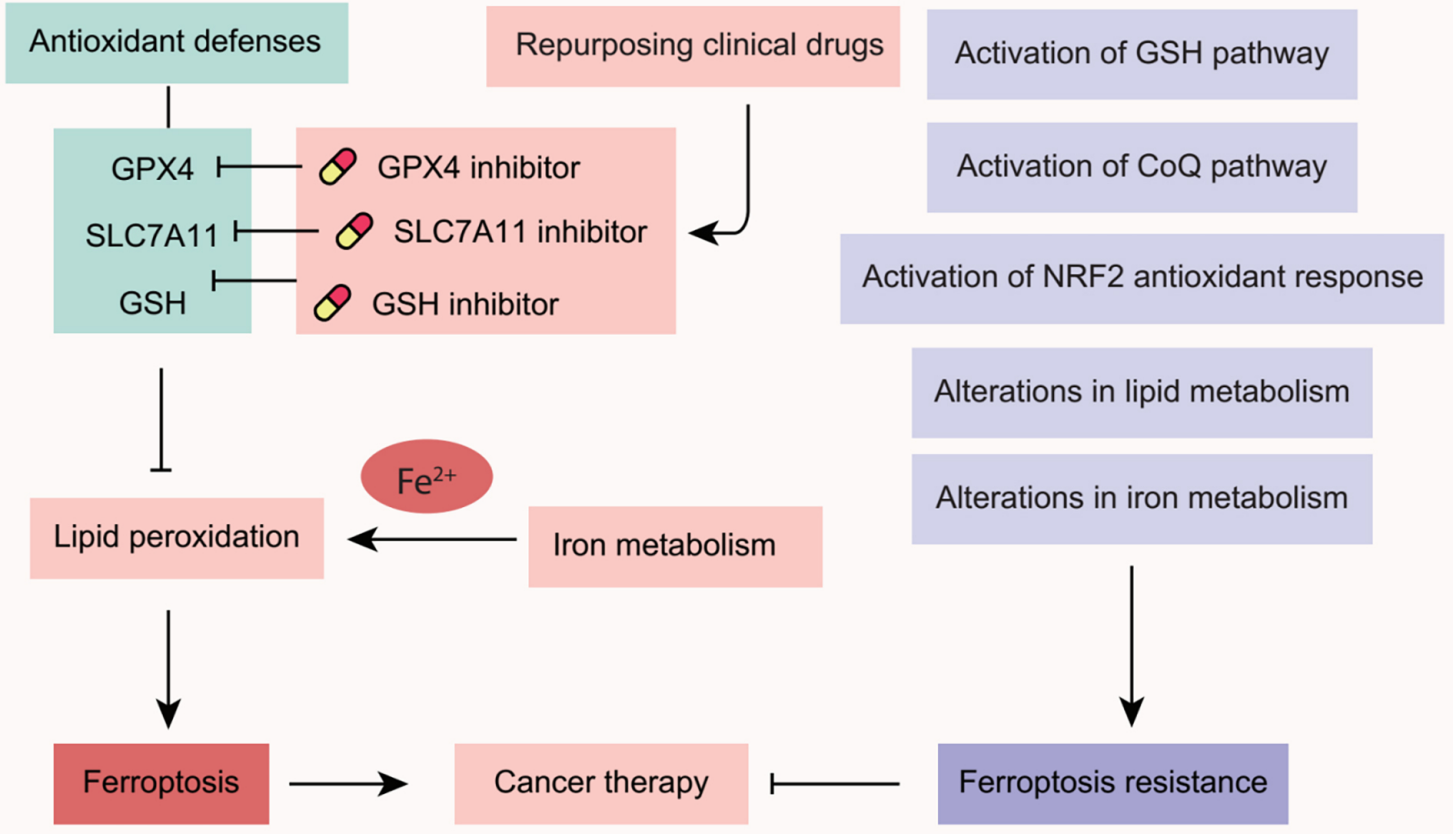
An overview of ferroptosis induction and ferroptosis resistance in cancer therapy. SLC7A11: Solute carrier family 7 member 11; GPX4: glutathione peroxidase 4; GSH: glutathione; NRF2: NFE2-like bZIP transcription factor 2; NFE2: nuclear factor, erythroid 2; CoQ: ubiquinone.

In recent years, applying ferroptosis to combat cancer has emerged as a focal point in etiological research and therapeutic development. Several tumor suppressors (e.g., p53 and Par-4) execute their tumor suppressive function in part by triggering ferroptosis^[7,8]^, suggesting that ferroptosis acts as a natural mechanism for tumor suppression. Notably, mesenchymal and dedifferentiated malignant cells, which frequently resist apoptotic cell death and conventional treatments, exhibit significant susceptibility to ferroptosis^[9,10]^. Consequently, targeting ferroptosis is considered a promising strategy for treating refractory tumors^[11]^. Additionally, ferroptosis-based interventions have demonstrated efficacy in overcoming resistance to traditional therapies and enhancing the effects of radiotherapy and immunotherapy^[12,13]^. However, cancer cells can develop various strategies to evade ferroptotic cell death, as discussed in this review [Figure 1]. Thus, a comprehensive understanding of the mechanisms underlying ferroptosis resistance in cancer may guide the development of effective cancer treatments.

In this review, we outline the fundamental molecular mechanisms underlying ferroptosis and summarize the application of various ferroptosis inducers in cancer treatment. In addition, we place particular emphasis on the crucial role of various signaling pathways in modulating ferroptosis resistance in cancer.

## MOLECULAR MECHANISMS OF FERROPTOSIS

**Figure 2:**
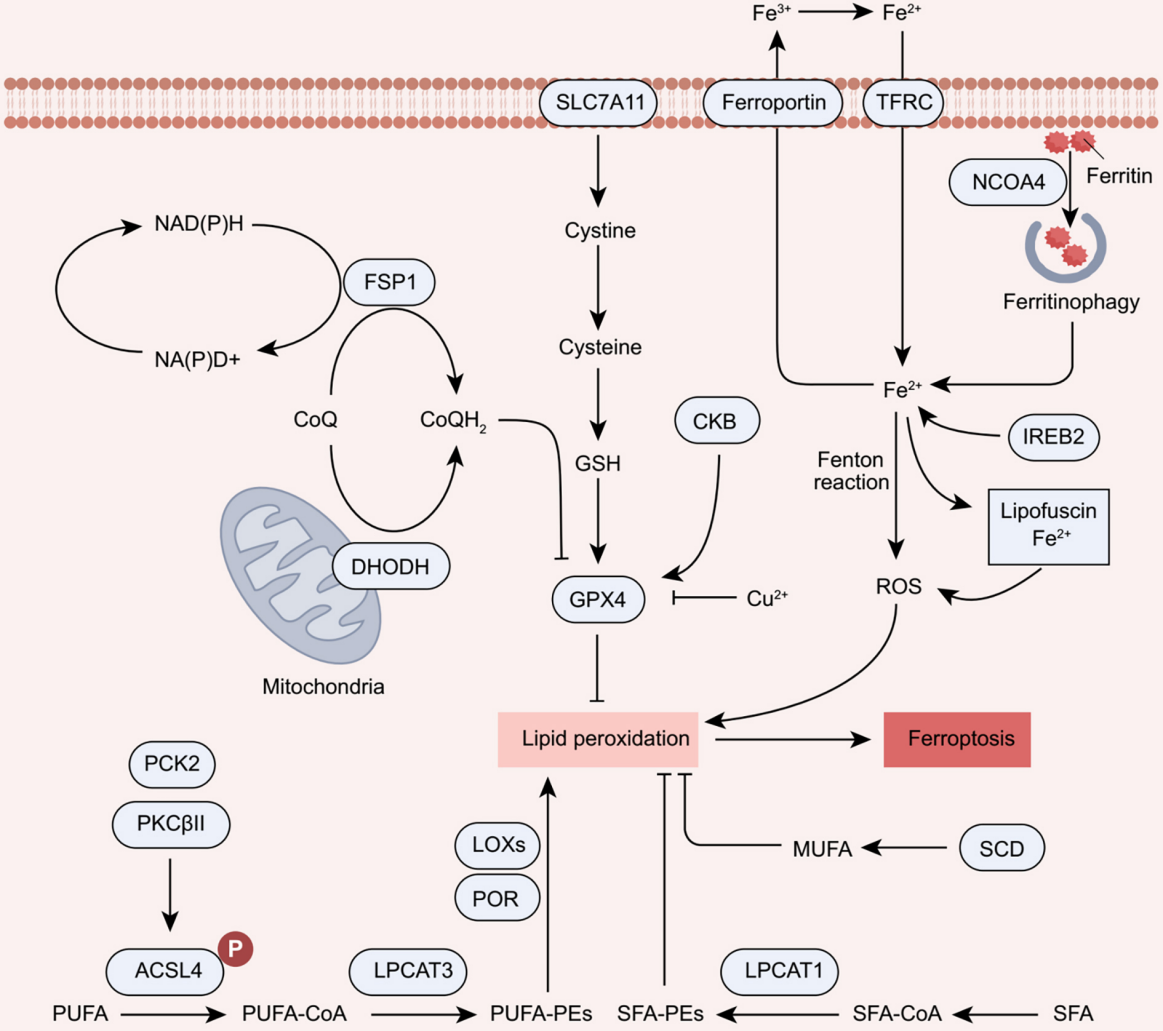
Molecular mechanisms of ferroptosis. Ferroptosis is an iron-dependent cell death characterized by increased lipid peroxidation. Ferroptosis involves an imbalance in the lipid peroxidation and antioxidant systems. IREB2, TFRC, and NCOA4-mediated ferritinophagy promote ferroptosis by increasing Fe^2+^ in cells, whereas ferroportin-mediated iron export inhibits ferroptosis. Subsequently, Fe^2+^ generates ROS through Fenton reaction. Furthermore, ACSL4, LPCAT3, LOXs, and POR pathways facilitate the peroxidation of PUFA, which is required for oxidative damage in ferroptosis. Meanwhile, PKCβII and PCK2 promote the phosphorylation of ACSL4, leading to enhanced phospholipid remodeling capability of ACSL4. Several antioxidant defense systems such as the SLC7A11-GSH-GPX4 and FSP1/DHODH-CoQ pathways play crucial roles in counteracting lipid peroxidation. Moreover, SCD and LPCAT1 inhibit ferroptosis by promoting MUFA or SFA-phospholipids synthesis. SFA: Saturated fatty acids; PE: phosphatidylethanolamine; SLC7A11: solute carrier family 7 member 11; TFRC: transferrin receptor; FSP1: ferroptosis suppressor protein-1; CKB: creatine kinase B; IREB2: iron-responsive element binding protein 2; DHODH: dihydroorotate dehydrogenase; GSH: glutathione; GPX4: glutathione peroxidase 4; ROS: reactive oxygen species; MUFA: monounsaturated fatty acid; PCK2: phosphoenolpyruvate carboxykinase 2; PKCβII: protein kinase CβII; ACSL4: acyl-CoA synthetase long-chain family member 4; POR: P450 oxidoreductase; LOXs: lipoxygenases; SCD: stearoyl-CoA desaturase; LPCAT3: lysophosphatidylcholine acyltransferase 3; LPCAT1: lysophosphatidylcholine acyltransferase 1; CoA: coenzyme A; CoQH2: dihydrouquinone; CoQ: ubiquinone.

Ferroptosis is caused by lipid peroxidation, a process in which free radicals react with carbon-carbon double bonds of lipids, leading to cell damage and death [Figure 2]. This oxidative process is closely linked to the dysregulation of antioxidant defenses that safeguard cells against lipid peroxidation. Additionally, it involves the accumulation of free iron, which produces reactive oxygen species (ROS) that exacerbate lipid peroxidation.

### Iron metabolism

Iron is essential to living organisms and plays a key role in various physiological processes, such as oxygen transport and enzyme catalysis^[14]^. Due to its ability to accept and provide electrons, iron cycles between Fe^2+^ and Fe^3+^ forms, allowing many non-heme- and heme-containing enzymes [e.g., Lipoxygenases (LOXs)] to function properly. The redox cycling ability of iron makes it versatile and widely used in numerous catalytic reactions, such as Fenton reaction. This process involves the reduction of hydrogen peroxide (H_2_O_2_) by Fe²^+^, producing hydroxyl radicals (•OH). These highly reactive radicals can attack PUFA in cellular membranes, initiating lipid peroxidation to induce ferroptosis^[15]^. Interestingly, the aging pigment lipofuscin can capture iron ions, which in turn promotes ferroptosis by generating ROS^[16]^. Consequently, iron chelators can potentially be used to modulate ferroptosis in diseases where it plays a pathogenic role^[1]^. In tumor cells, the availability of iron is tightly controlled at both the systemic and cellular levels to modulate ferroptosis^[17]^. The iron-responsive element binding protein 2 (IREB2) is a key regulator of iron homeostasis during ferroptosis by translationally regulating the expression of genes involved in iron metabolism^[1]^. The regulation of ferroptosis in cancer cells critically depends on the balance between transferrin receptor (TFRC)-mediated iron uptake, ferroportin-driven iron export, and ferritin-based iron storage^[18-20]^. As such, autophagy-mediated ferritin degradation (ferritinophagy) can lead to excessive free iron, ultimately triggering ferroptosis in cancer cells^[20-22]^.

### Lipid peroxidation

Lipid peroxides are produced by chemical reactions between lipids and oxygen. Peroxides formed on PUFAs within membrane phospholipids and their reactive aldehyde metabolites are thought to drive ferroptosis^[23-25]^. As a result, membranes with elevated PUFA-PL levels are particularly susceptible to peroxidation. Acyl-CoA synthetase long-chain family member 4 (ACSL4) acts as a key driver of ferroptosis^[23,24]^. The contribution of ACSL4 to ferroptosis relies on its capability to link PUFA (e.g., arachidonic acid and adrenoic acid) with coenzyme A (CoA) to form PUFA-CoA, which can be re-esterified in phospholipids by various Lysophosphatidylcholine acyltransferase (LPCAT) enzymes^[26]^. Lysophosphatidylcholine acyltransferase 3 (LPCAT3) promotes the induction of ferroptosis, while lysophosphatidylcholine acyltransferase 1 (LPCAT1) triggers ferroptosis evasion in cancer cells^[24,27]^. Notably, ACSL4 expression correlates with sensitivity to ferroptosis inducers in triple-negative breast cancer (TNBC), clear cell kidney cancer, and drug-resistant mesenchymal cancer cells^[9,23,28]^. Additionally, the phospholipid remodeling capability of ACSL4 is further amplified by phosphoenolpyruvate carboxykinase 2 (PCK2)- and protein kinase CβII (PKCβII)-mediated phosphorylation modification^[29,30]^. In contrast, acyl-CoA synthetase long-chain family member 3 (ACSL3) can either promote or inhibit ferroptotic cell death in a context-dependent manner^[31,32]^.

LOXs are iron-dependent dioxygenases that directly oxidize PUFA within biological membranes^[33]^. The ferroptosis-inducing role of LOXs is supported by the observation that pharmacological inhibition or genetic depletion of LOXs suppresses ferroptosis in cancer cells *in vitro*^[34,35]^. Despite this, the deletion of 15-lipoxygenase (15-LOX) fails to prevent glutathione peroxidase 4 (GPX4) knockout-induced ferroptosis in mouse models of acute kidney injury^[36]^. This could be attributed to compensatory mechanisms between different LOX enzymes or other lipid peroxidizing enzymes. For instance, cytochrome P450 oxidoreductase (POR) is also involved in the initiation of lipid peroxidation in multiple cancer lineages, potentially compensating for the role of LOXs^[37]^.

### Antioxidant defenses

GPX4 is a unique member of the GPX protein family, distinguished by its ability to reduce phospholipid hydroperoxide to phospholipid alcohol^[38]^. The genetic or pharmacological inhibition of GPX4 triggers excessive lipid peroxidation and potent ferroptosis *in vitro* and *in vivo*^[36,39]^. GPX4 exists in three isoforms with distinct subcellular localizations: cytoplasm, mitochondria, and nucleus. Both cytoplasmic and mitochondrial GPX4 likely play crucial roles in protecting against ferroptosis within their respective subcellular compartments, whereas the function of nuclear isoform remains to be further explored^[40]^. Furthermore, creatine kinase B (CKB)-mediated phosphorylation of GPX4 at Ser104 abrogates GPX4 degradation through chaperone-mediated autophagy, while the binding of copper ions to GPX4’s Cys107/148 facilitates its autophagic degradation^[41,42]^. However, whether CKB inhibition or copper supplementation can overcome ferroptosis resistance remains to be further investigated.

Tumor cells acquire cystine primarily through the system Xc-, in which cystine is subsequently reduced to cysteine in the cytoplasm^[43]^. Reduced glutathione (GSH), synthesized from cysteine, is an essential cofactor for the enzymatic activity of GPX4. Solute carrier family 7 member 11 (SLC7A11, also known as xCT) is the active transporter subunit of the system Xc-. The expression of SLC7A11 is frequently elevated in cancer cells and tissues, enabling cancer cells to increase intracellular GSH levels and counteract ferroptosis^[44]^. Accordingly, genetic or pharmacologic inhibition of SLC7A11 leads to GSH depletion, GPX4 inhibition, and ferroptosis in many types of cancer^[1,7,45]^. Thus, the SLC7A11-GSH-GPX4 axis is considered to be the major pathway in ferroptosis defense. However, some cancer cells can survive even after GPX4 inactivation, suggesting the existence of alternative mechanisms that confer resistance to ferroptosis^[9]^.

Ferroptosis suppressor protein-1 (FSP1), encoded by AIF family member 2 (AIFM2) gene, protects against ferroptosis through a mechanism independent of GPX4^[46,47]^. FSP1’s localization to the plasma membrane is necessary for its role in ferroptosis inhibition^[47]^. Functioning as an NAD(P)H-dependent oxidoreductase, FSP1 catalyzes the conversion of coenzyme Q (CoQ) to reduced coenzyme Q (CoQH2), which traps lipid peroxidation free radicals to inhibit ferroptosis^[48]^. Similarly, dihydroorotate dehydrogenase (DHODH), an enzyme involved in pyrimidine metabolism, can also reduce CoQ to CoQH2 in mitochondria^[40]^. Upon GPX4 inactivation, DHODH scavenges mitochondrial lipid peroxidation and safeguards cells from ferroptosis^[40]^. Given its central role in ferroptotic processes, understanding the collaborative mechanisms of the CoQ pathway across different components could reveal new therapeutic strategies for managing ferroptosis resistance in cancer cells.

## FERROPTOSIS INDUCERS FOR CANCER THERAPY

Recent studies have highlighted the advantages of ferroptosis induction in cancer treatment, notably in eradicating aggressive malignancies that are resistant to conventional therapies^[12,49]^. Various agents capable of triggering ferroptosis predominantly target the SLC7A11-GSH-GPX4 pathway, leading to excessive lipid peroxidation in cancer cells. In the following sections, we will introduce several common categories of ferroptosis inducers utilized in cancer treatment, including SLC7A11 and GPX4 inhibitors, as well as the repurposing of clinical drugs as ferroptosis inducers [Table 1].

**Table 1:** Ferroptosis inducers for cancer therapy

**Inducers**	**Mechanism/target**	**Tumor type**	**Phase**	**Refs.**
Erastin	SLC7A11 inhibition	Fibrosarcoma	Preclinical	[1,52]
IKE	SLC7A11 inhibition	Breast cancer	Preclinical	[53]
RSL3	GPX4 inhibition	Colorectal cancer	Preclinical	[39]
ML162	GPX4 inhibition	Fibrosarcoma	Preclinical	[55,56]
ML210	GPX4 inhibition	Pancreatic cancer, melanoma	Preclinical	[57]
Bufotalin	GPX4 degradation	NSCLC	Preclinical	[60]
DMOCPTL	GPX4 degradation	TNBC	Preclinical	[61]
N6F11	GPX4 degradation	Pancreatic cancer	Preclinical	[59]
PdPT	GPX4 degradation	NSCLC	Preclinical	[62]
Cu^2+^	GPX4 degradation	Pancreatic cancer	Preclinical	[42]
FIN56	GPX4 degradation	Fibrosarcoma	Preclinical	[63-65]
dGPX4	GPX4 degradation	Fibrosarcoma	Preclinical	[66]
GDC-11	GPX4 degradation	Fibrosarcoma	Preclinical	[67]
ZX703	GPX4 degradation	Fibrosarcoma	Preclinical	[68]
PDTACs	GPX4 degradation	NSCLC	Preclinical	[69]
Sulfasalazine	SLC7A11 inhibition	Colorectal cancer, esophageal cancer, TNBC	Approved antibiotics	[71-73]
Sorafenib	SLC7A11 inhibition	hepatocellular carcinoma	Approved anticancer drug	[75]
Cisplatin	GSH depletion	NSCLC, colorectal cancer	Approved anticancer drug	[78]
Artemisinins	ROS induction	Head and neck cancer, hepatocellular carcinoma, glioblastoma, lung cancer	Approved anti-malarial drugs	[82-86]

SLC7A11: Solute carrier family 7 member 11; GPX4: glutathione peroxidase 4; GSH: glutathione; ROS: reactive oxygen species; NSCLC: non-small cell lung cancer; TNBC: triple-negative breast cancer.

### SLC7A11 inhibitors

The inhibition of SLC7A11 induces the depletion of intracellular GSH, which in turn disrupts the antioxidant capacity of cells and decreases the enzyme activity of GPX4^[1]^. Consequently, SLC7A11 inhibitors cause excessive lipid peroxidation and ferroptosis in various cancer cells. Notably, certain types of cancer, such as Kirsten rat sarcoma viral oncogene (KRAS)-mutant lung adenocarcinomas and pancreatic cancer, are highly sensitive to the intervention targeting SLC7A11^[50,51]^. Erastin, a classic ferroptosis inducer, directly binds and inhibits the activity of SLC7A11, thereby inducing ferroptosis in cancer cells^[1,52]^. Although erastin exhibits excellent *in vitro* anticancer activity, it has low oral bioavailability due to poor water solubility^[53]^. Subsequently, an erastin derivative called imidazole ketone erastin (IKE) has been developed, which shows improved water solubility and potency in animal models^[53]^. Moreover, other studies have identified several SLC7A11 inhibitors through high-throughput virtual screening, but the specificity of these agents in inducing ferroptosis requires further validation^[54]^.

### GPX4 inhibitors

Certain small-molecule agents, including RSL3, ML162, and ML210, can target the nucleophilic active sites of GPX4 to trigger ferroptosis in cancer cells. RSL3, initially recognized as a compound selectively lethal to oncogenic RAS-expressing cells, was later identified as a GPX4 inhibitor^[39]^. The effectiveness of RSL3 relies on its chloroacetamide moiety, which targets the nucleophilic selenocysteine residue on GPX4, thereby inhibiting GPX4 activity^[39]^. Similarly, ML162 has an activated alkyl chloride that inhibits GPX4 activity by covalently binding to Sec46 and Cys66 of GPX4^[55,56]^. However, ML210 is a covalent GPX4 inhibitor with a functional nitroisoxazole moiety, thus operating through a mechanism distinct from RSL3 and ML162^[57]^. As a prodrug, ML210 transforms within cells into the corresponding α-nitroketoxime JKE-1674, which specifically binds to GPX4^[57]^. While RSL3 and ML162 suppress tumor growth and decrease GPX4 expression in 3D spheroid tumor models, ML210 is ineffective in this context^[58]^. Furthermore, although these GPX4 inhibitors exhibit strong antitumor effects *in vitro*, there is limited evidence for their antitumor efficacy *in vivo*.

GPX4 protein degraders can also serve as ferroptosis inducers for cancer treatment. Several compounds, such as bufotalin, DMOCPTL, N6F11, and PdPT, trigger ferroptosis by inducing proteasome-mediated degradation of GPX4 in pancreatic, lung, and TNBC cells *in vitro* and *in vivo*^[59-62]^. Copper ions increase the autophagic degradation of GPX4 by directly binding to Cys107/148 of GPX4 protein, leading to ferroptosis in pancreatic cancer cells^[42]^. FIN56, a ferroptosis-inducing agent, functions via its oxime and piperidine and promotes GPX4 degradation through either proteasome or autophagy pathways in cancer cells, depending on the cellular context^[63-65]^. In addition, targeted protein degradation by proteolysis targeting chimeras (PROTACs) is a novel therapeutic strategy for inducing ferroptosis in cancer cells. PROTAC-based GPX4 degraders (e.g., dGPX4, GDC-11, and ZX703) are usually composed of a GPX4 inhibitor (such as ML162 or ML210), pomalidomide, and a linking moiety^[66-68]^. The warhead of ML162 or ML210 can bind to GPX4 protein, and promalidomide can recruit the ubiquitin E3 ligase. However, the *in vivo* application of PROTAC-based GPX4 degraders is limited by their poor water solubility and low stability^[66]^. Furthermore, photodegradation-targeting chimeras (PDTACs)-based GPX4 degrader contains the light-sensitive component verteporfin, which produces ROS upon light exposure, and possesses the potential to induce ferroptosis^[69]^. PDTAC-induced GPX4 degradation, which occurs after light exposure, does not require intracellular degradation machineries such as proteasomes and lysosomes^[69]^. However, the factors determining whether GPX4 is destructed by ubiquitin-proteasome system or autophagy remains largely unclear.

### Repurposing clinical drugs as ferroptosis inducers

Several clinically used drugs, such as sulfasalazine, sorafenib, cisplatin, and artemisinins, have demonstrated ferroptosis-inducing properties. Originally used to treat inflammatory bowel diseases and rheumatoid arthritis, sulfasalazine has also been investigated for its potential to act as a ferroptosis inducer^[70]^. It has been reported to induce ferroptosis by repressing SLC7A11 in various cancer cells, including colorectal cancer, esophageal cancer, and TNBC^[71-73]^. Sorafenib, a multi-kinase inhibitor used for advanced cancers, is thought to induce ferroptosis by targeting SLC7A11^[74]^. However, recent research reveals that sorafenib is ineffective at inducing ferroptosis in various cancer cell lines^[75]^, highlighting the need for strategies to improve its specificity for ferroptosis induction. In line with this insight, JB3, a sorafenib derivative, was designed and showed high oral bioavailability and ferroptosis-inducing ability in xenograft models^[76]^. Platinum compounds are widely used anticancer drugs and trigger cancer cell death primarily by triggering DNA damage^[77]^. Cisplatin has been shown to induce ferroptosis by depleting intracellular GSH in several types of cancer cells, including non-small cell lung cancer (NSCLC) and colorectal cancer^[78]^. Accordingly, the combination of cisplatin with erastin offers a potential therapeutic approach for overcoming tumor resistance to cisplatin^[79,80]^. Artemisinins, extracted from *Artemisia annua*, have well-known anti-malarial effects and promising antitumor activities^[81]^. Artemisinins have been shown to trigger ferroptosis in head and neck cancer, lung cancer, hepatocellular carcinoma, and glioblastoma cells^[82-86]^. Mechanistically, artemisinins elevate intracellular Fe^2+^ through both ferritinophagy-dependent and -independent mechanisms, thereby promoting lipid peroxidation and inducing ferroptosis ^[87]^. Collectively, these findings suggest a potential therapeutic avenue for utilizing ferroptosis-inducing drugs in cancer treatment.

Gene editing technology and RNA-based therapies also hold considerable promise for promoting ferroptosis in cancer cells, thus advancing anticancer strategies. For instance, using small interfering RNAs (siRNAs) or short hairpin RNAs (shRNA) to downregulate GPX4 or SLC7A11 could sensitize tumor cells to ferroptosis and inhibit tumor growth^[1,39]^. Similarly, anti-miRs and miRNA mimics could modulate the expression of ferroptosis-associated genes, which could facilitate targeted induction of ferroptosis in cancer cells^[88]^. In addition, combining RNA-based therapies with ferroptosis inducers could present a promising approach to cancer treatment.

## SIGNALING INVOLVED IN FERROPTOSIS RESISTANCE IN CANCER

Despite the potential of ferroptosis in cancer treatment, some cancer cells exhibit resistance to ferroptosis-inducing agents. Deciphering the mechanisms underlying ferroptosis resistance is critical for enhancing the efficacy of ferroptosis-based therapies. Below, we will introduce the mechanisms of ferroptosis resistance from several perspectives, including the activation of the GSH pathway, the CoQ pathway, and NFE2-like bZIP transcription factor 2 (NRF2) antioxidant response, as well as the alterations in lipid and iron metabolism [Table 2].

**Table 2:** Modulators of ferroptosis resistance in cancer

**Genes**	**Effects on ferroptosis**	**Model**	**Mechanism**	**Refs.**
**GSH pathway**				
ZRANB1	Pro-ferroptosis	Renal cancer	Enhances SLC7A11 degradation	[99]
GGT1	Anti-ferroptosis	Glioblastoma	Increases cysteine	[89]
ZDHHC8	Anti-ferroptosis	Glioblastoma	Enhances SLC7A11 stabilization	[100]
USP20	Anti-ferroptosis	Hepatocellular carcinoma	Enhances SLC7A11 stabilization	[101]
CBS	Anti-ferroptosis	Pancreatic cancer	Enhances cysteine synthesis	[126]
NEDD4L	Pro-ferroptosis	NSCLC	Enhances GPX4 degradation	[102]
STK33	Anti-ferroptosis	Pancreatic cancer	Inhibits GPX4 degradation	[103]
HER2	Anti-ferroptosis	Breast cancer	Enhances GPX4 expression	[104]
c-Fos	Pro-ferroptosis	Colorectal cancer	Decreases SLC7A11 expression	[91]
SF3B1	Anti-ferroptosis	Lung cancer	Enhances SLC7A11 expression	[93]
SOX2	Anti-ferroptosis	Lung cancer	Enhances SLC7A11 expression	[92]
ATF4	Anti-ferroptosis	Renal cell carcinoma	Enhances SLC7A11 expression	[95]
NSUN2	Anti-ferroptosis	Endometrial cancer	Enhances SLC7A11 stabilization	[96]
METTL3	Anti-ferroptosis	Hepatoblastoma	Enhances SLC7A11 mRNA stabilization	[97]
mTORC1	Anti-ferroptosis	Laryngeal squamous cell carcinoma	Enhances SLC7A11 expression	[94]
TCF4	Anti-ferroptosis	Gastric cancer	Enhances GPX4 expression	[105]
NeuroD1	Anti-ferroptosis	Hepatocellular carcinoma	Enhances GPX4 expression	[106]
**CoQ pathway**				
CEBPB	Anti-ferroptosis	Pancreatic cancer	Enhances FSP1 mRNA stabilization	[109]
ALDH1A3	Anti-ferroptosis	Colorectal cancer	Enhances CoQ synthesis	[115]
POLQ	Anti-ferroptosis	Gastric cancer	Enhances DHODH expression	[112]
PRR11	Anti-ferroptosis	Gliomas	Enhances the stabilization of DHODH	[113]
NRF2	Anti-ferroptosis	Lung cancers	Enhances FSP1 expression	[108]
TRIM21	Anti-ferroptosis	Hepatocellular carcinoma, pancreatic cancer	Enhances FSP1 plasma membrane translocation	[110]
ACSL1	Anti-ferroptosis	Ovarian cancer	Decreases FSP1 degradation	[111]
**NRF2 antioxidant response**				
Src	Anti-ferroptosis	Glioblastoma	Enhances NRF2 activity	[121]
ATF3	Anti-ferroptosis	Gastric carcinoma	Enhances NRF2 activity	[128]
CYBB	Anti-ferroptosis	Mesenchymal glioblastoma	Enhances NRF2 activity	[127]
CBS	Anti-ferroptosis	Ovarian cancer	Acts as NRF2 target gene, enhances cysteine synthesis	[126]
SLC7A11	Anti-ferroptosis	Renal cell carcinoma	Acts as NRF2 target gene, enhances GSH synthesis	[120]
DPP9	Anti-ferroptosis	Clear cell renal cell carcinoma	Increases NRF2 stabilization	[120]
ABCC5	Anti-ferroptosis	Hepatocellular carcinoma	Acts as NRF2 target gene, decreases ROS	[124]
MT1G	Anti-ferroptosis	Hepatocellular carcinoma	Acts as NRF2 target gene, decreases ROS	[125]
NSUN2	Anti-ferroptosis	NSCLC	Enhances NRF2 expression	[122]
**Lipid metabolism**				
SCD5	Anti-ferroptosis	NSCLC	Enhances *de novo* MUFA synthesis	[134]
SCD1	Anti-ferroptosis	Colorectal cancer, melanoma	Enhances *de novo* MUFA synthesis	[131,133]
SLC27A4	Anti-ferroptosis	Hepatocellular carcinoma	Enhances uptake of MUFA	[135]
ACSM1/3	Anti-ferroptosis	Prostate cancer	Enhances fatty acid oxidation	[137]
PDK4	Anti-ferroptosis	Pancreatic cancer	Hinders pyruvate oxidation and fatty acid synthesis	[136]
15-LOX	Pro-ferroptosis	Cervical cancer	Enhances lipid synthesis	[143]
B7H3	Anti-ferroptosis	Colorectal cancer	Decreases cholesterol metabolism	[141]
27HC	Anti-ferroptosis	Breast cancer	Decreases cholesterol metabolism	[140]
**Iron metabolism**				
circRAPGEF5	Anti-ferroptosis	Endometrial cancer	Decreases iron uptake	[150]
TRIM33	Pro-ferroptosis	Hepatocellular carcinoma	Decreases TFRC stabilization	[151]
METTL16	Anti-ferroptosis	Hepatocellular carcinoma	Enhances LTF stabilization	[152]
DUSP4	Anti-ferroptosis	Hepatocellular carcinoma	Enhances FTH and FTL expression	[156]
SOX13	Anti-ferroptosis	Gastric cancer	Decreases mitochondrial ROS	[154]
RBCK1	Anti-ferroptosis	Pancreatic cancer	Decreases mitochondrial ROS	[155]
TRPML1	Anti-ferroptosis	Breast cancer, NSCLC	Decreases intracellular iron	[159]
PROM2	Anti-ferroptosis	Bladder cancer, breast cancer, lung cancer	Enhances iron export	[146-149]

SLC7A11: Solute carrier family 7 member 11; GPX4: glutathione peroxidase 4; GSH: glutathione; ROS: reactive oxygen species; NSCLC: non-small cell lung cancer; TNBC: triple-negative breast cancer; FTH: ferritin heavy chain; FTL: ferritin light chain; LTF: lactotransferrin; TFRC: transferrin receptor; MUFA: monounsaturated fatty acid; NRF2: NFE2-like bZIP transcription factor 2; NFE2: nuclear factor, erythroid 2; FSP1: ferroptosis suppressor protein-1; DHODH: dihydroorotate dehydrogenase; CBS: cystathionine beta-synthase.

### Activation of the GSH pathway

**Figure 3:**
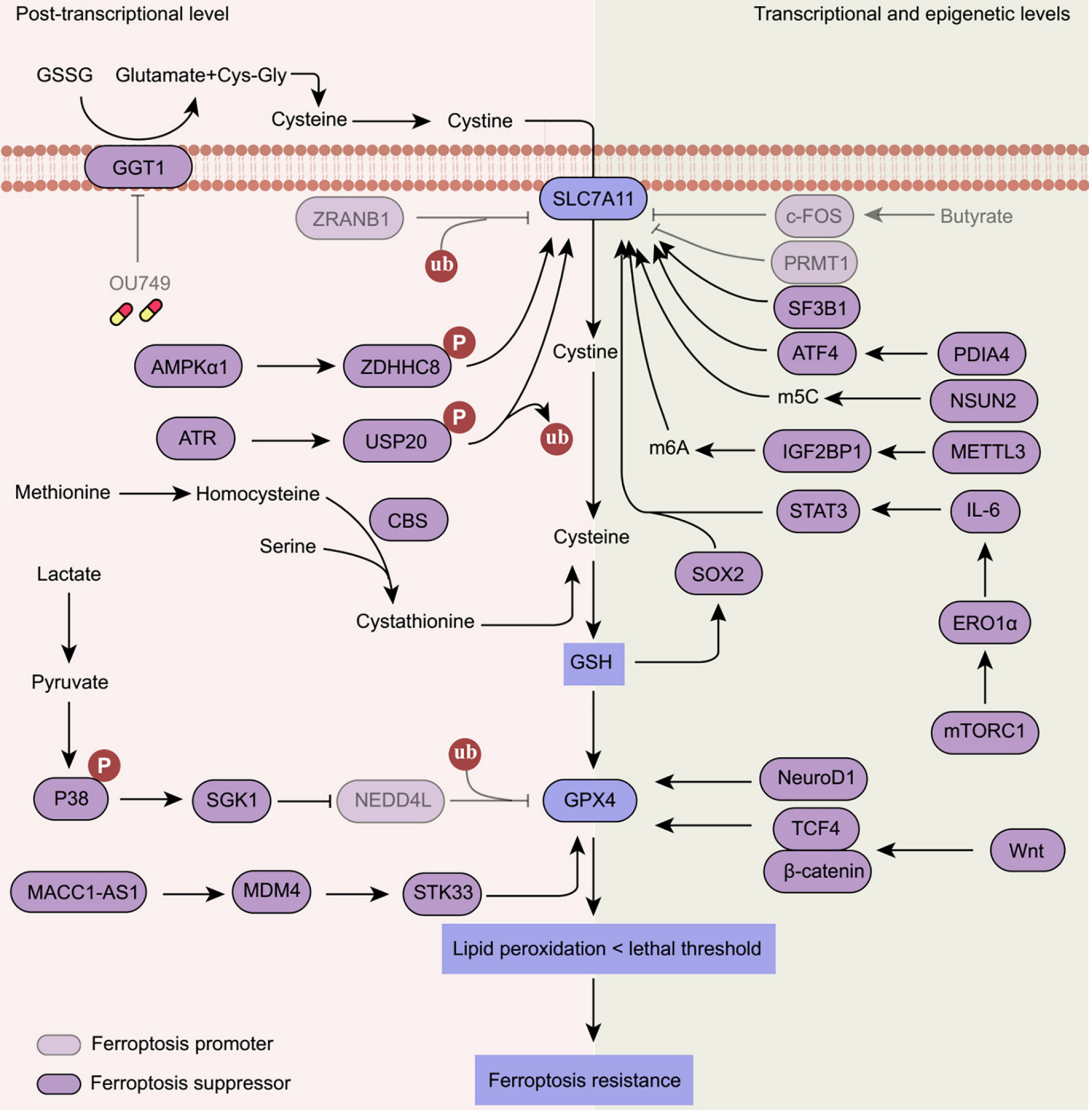
Ferroptosis resistance induced by the activation of the GSH pathway. SLC7A11-GSH-GPX4 axis is regulated at the transcriptional, epigenetic, and post-transcriptional levels. The protein expression or activity of SLC7A11 is regulated by ZRANB1, ZDHHC8, USP20, c-FOS, PRMT1, SF3B1, ATF4, NSUN2, IGF2BP1, STAT3, and SOX2. In addition, GPX4 is regulated by NEDD4L, STK33, NeuroD1, and TCF4. OU749, a GGT1 inhibitor. SLC7A11: Solute carrier family 7 member 11; GPX4: glutathione peroxidase 4; GSH: glutathione; CBS: cystathionine beta-synthase; GGT1: gamma-glutamyltransferase 1; STAT3: signal transducer and activator of transcription 3; IL-6: interleukin 6; ZRANB1: zinc finger RANBP2-type containing 1; ZDHHC8: zinc finger DHHC-type palmitoyltransferase 8; USP20: ubiquitin-specific peptidase 20; SF3B1: splicing factor 3b subunit 1; ATF4: activating transcription factor 4; NSUN2: NOP2/Sun RNA methyltransferase 2; SOX2: SRY-box transcription factor 2; NEDD4L: NEDD4 like E3 ubiquitin protein ligase; STK33: serine/threonine kinase 33; TCF4: transcription factor 4; NeuroD1: neuronal differentiation 1; METTL3: methyltransferase-like protein 3; PDIA4: protein disulfide isomerase family A member 4; PRMT1: protein arginine methyltransferase 1.

The GSH-SLC7A11-GPX4 axis serves as the primary defense against ferroptosis and is regulated at multiple levels through diverse mechanisms [Figure 3].

Lipid peroxidation is normally suppressed to a large extent by GPX4, which requires GSH as a key cofactor^[1,39]^. The dysregulation of cystine or GSH metabolism may contribute to ferroptosis resistance in cancer cells. Gamma-glutamyltransferase 1 (GGT1) facilitates the conversion of extracellular GSH into cysteinyl-glycine, which is further degraded into cysteine. Notably, GGT1 decreases the susceptibility of glioblastoma cells to ferroptosis triggered by cystine deprivation, particularly under conditions of high cell density^[89]^. Therefore, the combination of GGT1 inhibitors with ferroptosis inducers holds significant potential as a promising therapeutic strategy for glioblastoma^[89]^. In addition, cancer-associated fibroblasts participate in pancreatic cancer tumorigenesis by secreting cysteine, which confers cancer resistance to ferroptosis^[90]^. In cancer-associated fibroblasts, cystathionine beta-synthase (CBS)-dependent transsulfuration pathway supports cysteine synthesis, thereby inducing ferroptosis resistance in pancreatic cancer^[90]^.

Emerging insights indicate that SLC7A11 may serve as a pivotal factor of failure in ferroptosis induction. SLC7A11 expression is higher in oxaliplatin-resistant colorectal cancer cells compared to those sensitive to oxaliplatin^[91]^. This resistance correlates with the reduced levels of butyrate, the most prevalent microbial fermentation product in the colon, commonly observed in colorectal cancer patients^[91]^. Mechanistically, butyrate inhibits SLC7A11 expression and GSH synthesis by inducing the expression of the transcription factor c-Fos^[91]^. Therefore, combining butyrate with oxaliplatin may be an effective way to reduce ferroptosis resistance^[91]^. SLC7A11 can be activated by the transcriptional factor SRY-box transcription factor 2 (SOX2) and the splicing factor 3b subunit 1 (SF3B1) in lung cancer cells^[92,93]^. In human lung cancer tissues, the overexpression of SOX2 and SF3B1 enhances cell resistance to ferroptosis and correlates positively with SLC7A11 expression^[92,93]^. Moreover, SLC7A11 is essential for the mechanistic target of rapamycin complex 1 (mTORC1)-mediated ferroptosis resistance. Aberrantly activated mTORC1 drives resistance to ferroptosis by promoting the transcription of SLC7A11 through stimulation of the endoplasmic reticulum stress-interleukin 6 (IL-6)- signal transducer and activator of transcription 3 (STAT3) pathway^[94]^. Similarly, the overexpression of protein disulfide isomerase family A member 4 (PDIA4) in the endoplasmic reticulum enhances the expression of the transcription factor activating transcription factor 4 (ATF4), which promotes the transcriptional expression of SLC7A11, thereby promoting ferroptosis resistance in renal cell carcinoma^[95]^.

The epigenetic modulation of SLC7A11 proteins has also been connected to ferroptosis resistance. For instance, 5-methylcytosine (m5C) and N6-methyladenosine (m6A) modifications have been identified to upregulate SLC7A11 mRNA in cancer cells, leading to ferroptosis resistance. Specifically, the m5C methyltransferase NOP2/Sun RNA methyltransferase 2 (NSUN2) catalyzes the m5C modification of SLC7A11 mRNA, thereby enhancing its stability in endometrial cancer^[96]^. In addition, the m6A methyltransferase methyltransferase-like protein 3 (METTL3)-dependent m6A modification increases SLC7A11 mRNA stability, thus inhibiting ferroptosis in hepatoblastoma^[97]^. Further progress in the understanding of how epigenetic mechanisms influence SLC7A11 expression may potentially identify novel therapeutic targets for cancer cells that are resistant to ferroptosis.

In addition to mRNA levels, elevated SLC7A11 protein stability likewise results in ferroptosis tolerance. The ubiquitin-proteasome system has essential roles in maintaining protein stability and has been implicated in the process of ferroptosis^[98]^. Loss of zinc finger RANBP2-type containing 1 (ZRANB1), an E3 ubiquitin ligase responsible for SLC7A11 protein degradation, renders renal cancer cells resistant to ferroptosis^[99]^. In glioblastoma, the S-palmitoylated modification of SLC7A11 is essential for its protein stabilization. Specifically, the palmitoyl transferase zinc finger DHHC-type palmitoyltransferase 8 (ZDHHC8) catalyzes S-palmitoylation of SLC7A11 at Cys327, thus increasing the deubiquitination of SLC7A11^[100]^. Consequently, ZDHHC8-mediated SLC7A11 stabilization fosters ferroptosis resistance during glioblastoma tumorigenesis^[100]^. Similarly, the deubiquitinase ubiquitin-specific peptidase 20 (USP20) stabilizes SLC7A11 via removing K48-specific ubiquitination of SLC7A11 protein at Lys30/37^[101]^. Thus, high expression of USP20 is associated with poor prognosis in hepatocellular carcinoma and contributes to oxaliplatin and ferroptosis resistance of hepatocellular carcinoma cells^[101]^.

Elevated expression of GPX4, which leads to increased lipid peroxidation detoxification capacity, is regarded as a key factor for ferroptosis resistance. In NSCLC cells, ferroptosis is implicated in etoposide-induced cell death. Lactate, a glycolytic metabolite, induces ferroptosis resistance by upregulating GPX4, leading to insensitivity to etoposide therapy^[102]^. Mechanistically, lactate inhibits the ubiquitination of GPX4 and enhances its stability through activation of the E3 ubiquitin ligase NEDD4 like E3 ubiquitin protein ligase (NEDD4L), thereby triggering ferroptosis and etoposide resistance^[102]^. Resistance to ferroptosis may also impact the efficacy of gemcitabine, which is a first-line chemotherapy drug for pancreatic cancer treatment^[103]^. The long non-coding RNA MACC1 antisense RNA 1 (MACC1-AS1) promotes the expression of serine/threonine kinase 33 (STK33) protein kinase, which inhibits GPX4 degradation, therefore counteracting the cytotoxic effect of gemcitabine^[103]^. GPX4 upregulation induced by the activation of erb-b2 receptor tyrosine kinase 2 (HER2 or ERBB2) pathway may contribute to ferroptosis resistance in luminal breast cancer cell lines^[104]^. In contrast, neratinib, an irreversible HER2 inhibitor, effectively reverses ferroptosis-resistant luminal breast cancer^[104]^. Moreover, the combination of neratinib with GPX4 inhibitors promotes ferroptosis by increasing the generation of mitochondria ROS and lipid peroxidation^[104]^. The transcription factor 4 (TCF4) and neuronal differentiation 1 (NeuroD1) can directly stimulate GPX4 transcription, thus inducing ferroptosis resistance in gastric cancer and hepatocellular carcinoma, respectively^[105,106]^. These findings suggest the development of clinical therapeutic strategies targeting the GSH pathway aimed at overcoming chemotherapy resistance in tumors.

### Activation of the CoQ pathway

**Figure 4:**
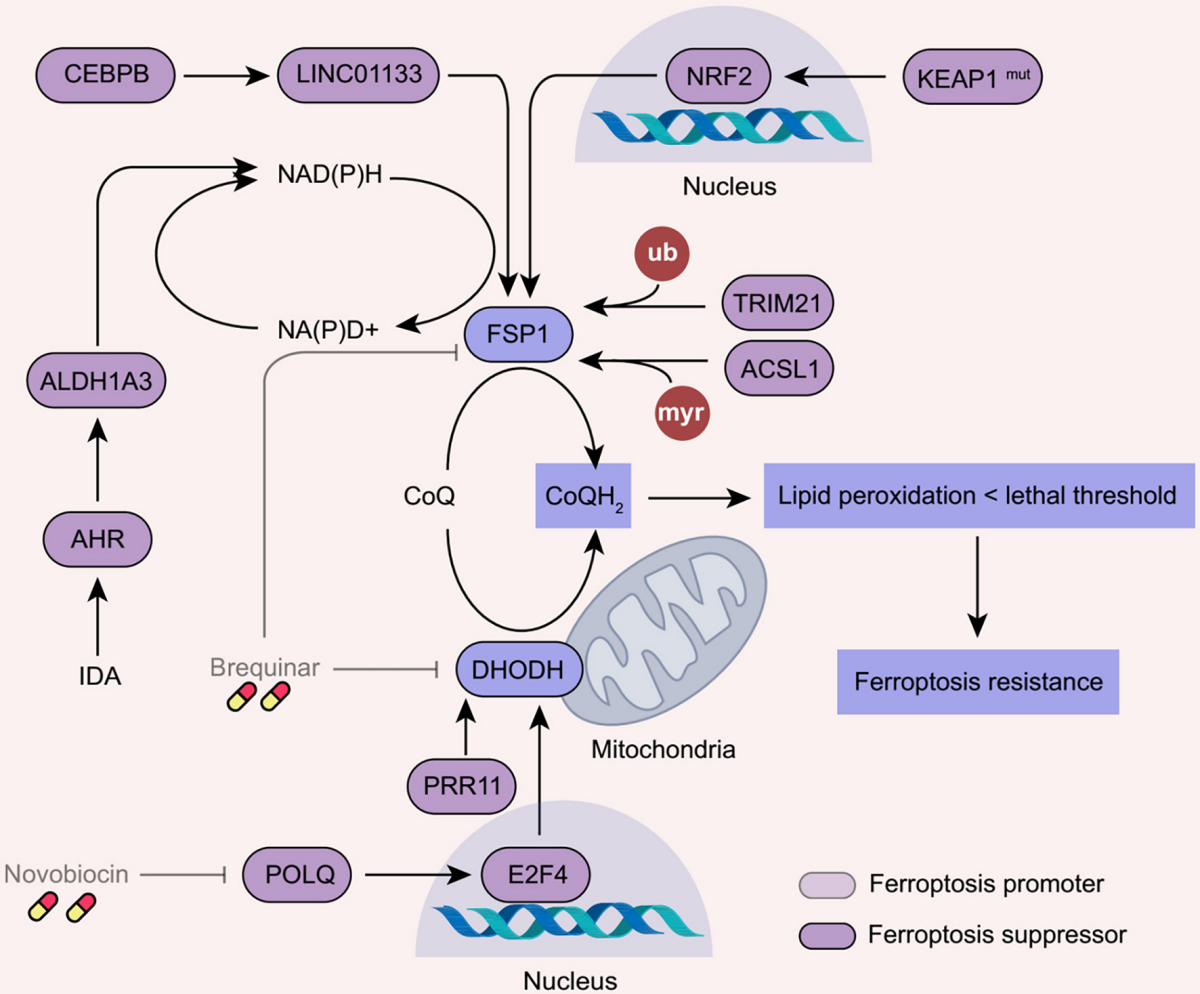
Ferroptosis resistance induced by the activation of the CoQ pathway. FSP1- or DHODH-mediated generation of CoQH_2_ functions as a key component in cellular antioxidant systems, contributing to ferroptosis resistance in cancer cells. Brequinar, a dual inhibitor of FSP1 and DHODH; Novobiocin, a POLQ inhibitor. FSP1: Ferroptosis suppressor protein-1; DHODH: dihydroorotate dehydrogenase. POLQ: polymerase theta; NRF2: NFE2-like bZIP transcription factor 2; NFE2: nuclear factor, erythroid 2; ACSL1: acyl-CoA synthetase long-chain family member 1; AHR: aryl hydrocarbon receptor; ALDH1A3: aldehyde dehydrogenase 1 family member A3; TRIM21: tripartite motif-containing 21; KEAP1: kelch-like ECH-associated protein 1; PRR11: proline-rich protein 11; CEBPB: CCAAT enhancer binding protein beta; PRR11: proline-rich protein 11; E2F4: E2F transcription factor 4.

CoQ is a key electron carrier in the ETC and also an integral component of the antioxidant system. FSP1 and DHODH can reduce CoQ to CoQH2, a lipophilic antioxidant^[40,46]^, thereby conferring resistance to ferroptosis in tumors [Figure 4].

FSP1 could be a critical player that mediates ferroptosis resistance in multiple tumors. In kelch-like ECH-associated protein 1 (KEAP1)-mutant lung cancers, NRF2 has been shown to promote the transcriptional expression of FSP1^[107,108]^. Additionally, CCAAT/enhancer-binding protein beta (C/EBPB) acts as a transcription factor to increase the expression of LINC01133, which stabilizes FSP1 mRNA in pancreatic cancer cells^[109]^. Thus, synergistically targeting of FSP1 and NRF2 may offer a maximal antitumor strategy for inducing ferroptosis in KEAP1-mutant tumors^[108]^. Furthermore, post-translational modifications, such as ubiquitination and N-myristoylation, modulate FSP1 activity and stability. For instance, the E3 ubiquitin ligase tripartite motif-containing 21 (TRIM21) acts as a suppressor of ferroptosis by promoting K63 ubiquitination and plasma membrane translocation of FSP1, making it a potential therapeutic target to enhance chemosensitivity in ferroptosis-resistant hepatocellular carcinoma and pancreatic cancer^[110]^. In addition, acyl-CoA synthetase long chain family member 1 (ACSL1), a regulator of fatty acid metabolism, inhibits the degradation of FSP1 by increasing its N-myristoylation, thereby antagonizing ferroptosis in ovarian cancer^[111]^. Therefore, the FSP1-CoQ pathway drives ferroptosis resistance and offers promising therapeutic targets for cancer treatment.

Similarly to FSP1, the activation of DHODH-CoQ pathways also contributes to ferroptosis resistance. Polymerase theta (POLQ), a DNA polymerase, drives resistance to ferroptosis in gastric cancer cells by stimulating DHODH expression via the transcription factor E2F transcription factor 4 (E2F4)^[112]^. The combination of POLQ inhibitor and ferroptosis inducer has synergistic inhibitory effects on gastric cancer stem cells, providing a potential clinically feasible strategy for gastric cancer^[112]^. Furthermore, the resistance to ferroptosis in recurrent gliomas is predominantly driven by proline-rich protein 11 (PRR11), which maintains DHODH protein stability^[113]^. Notably, DHODH inhibitors, such as brequinar, can also inhibit FSP1, potentially enhancing the sensitization of cancer cells to ferroptosis^[114]^. However, the precise mechanisms of crosstalk between FSP1 and DHODH are yet to be elucidated.

Nicotinamide adenine dinucleotide (NADH)-dependent redox balance is thought to contribute to CoQ-mediated ferroptosis resistance phenotypes. Activation of aryl hydrocarbon receptor (AHR) induces the expression of aldehyde dehydrogenase 1 family member A3 (ALDH1A3), which mediates ferroptosis resistance via producing reduced NADH, thus increasing the synthesis of CoQ^[115]^. As such, gut microbial metabolite trans-3-indoleacrylic acid directly activates AHR to increase ferroptosis resistance in colorectal cancer^[115]^. Consistently, targeted inhibition of the mevalonate pathway disturbs the redox balance within the CoQ pathway, thereby overcoming ferroptosis resistance in TNBC^[116]^. Additionally, the CoQ pathway participates in the development of radioresistance and presents a potential target for reversing radioresistance in NSCLC^[117]^. In contrast, statin treatment may enhance radiotherapy-mediated ferroptosis by disrupting CoQ synthesis^[117]^.

### Activation of NRF2 antioxidant response

**Figure 5:**
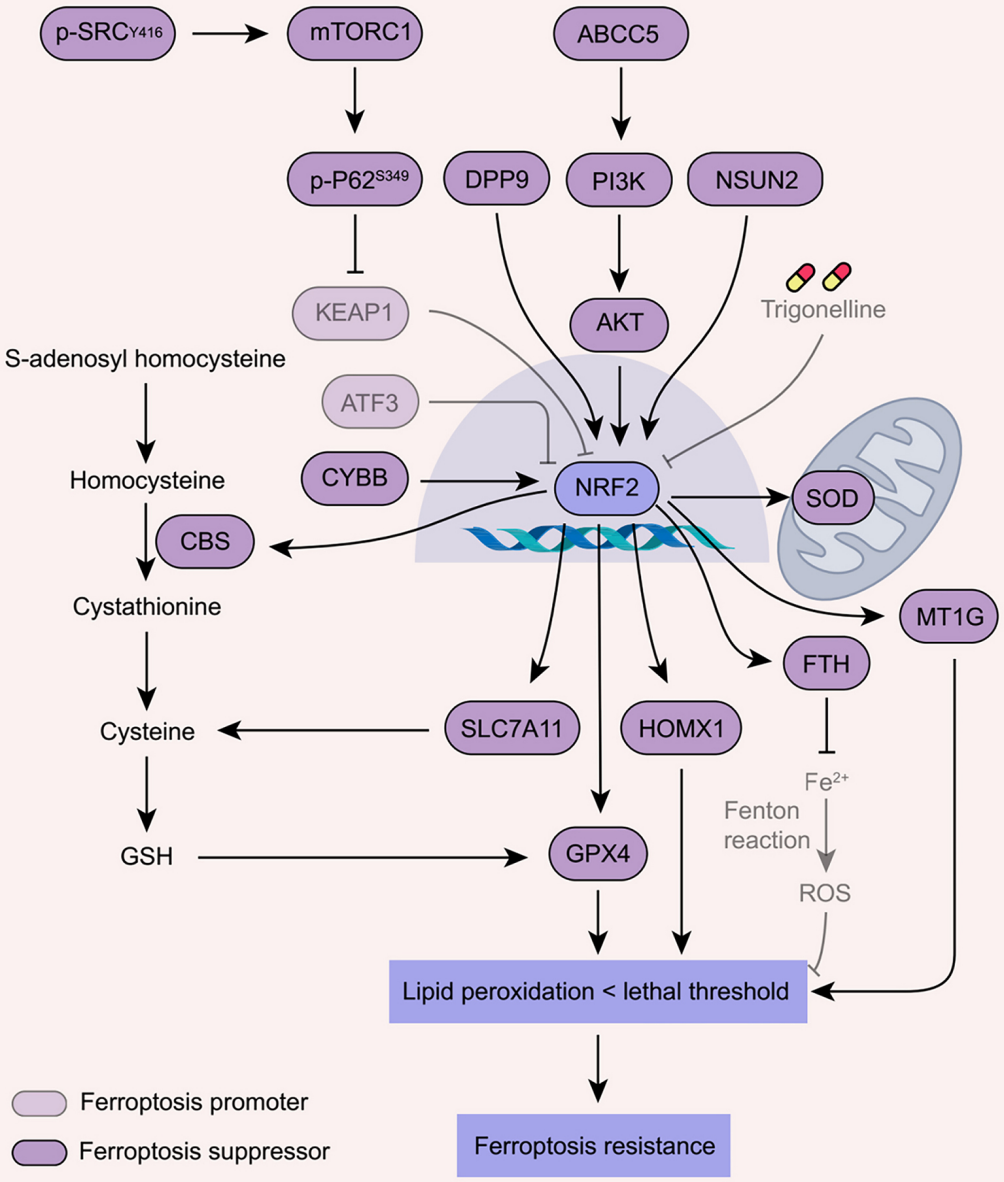
Ferroptosis resistance induced by the activation of NRF2 antioxidant response. NRF2 protein expression is modulated by CYBB, ATF3, p-SRC, DPP9, ABCC5, and NSUN2. Moreover, NRF2 promotes ferroptosis resistance by transcriptionally upregulating several target genes, including CBS, SLC7A11, GPX4, HOMX1, FTH, MT1G, and SOD. SLC7A11: Solute carrier family 7 member 11; GPX4: glutathione peroxidase 4; GSH: glutathione; CYBB: cytochrome b-245 beta chain; CBS: cystathionine beta-synthase; FTH: ferritin heavy chain; SOD: superoxide dismutase; ABCC5: ATP binding cassette subfamily C member 5; MT1G: metallothionein 1G; DPP9: dipeptidyl peptidase 9; ATF3: activating transcription factor 3; NSUN2: NOP2/Sun RNA methyltransferase 2; NRF2: NFE2-like bZIP transcription factor 2; NFE2: nuclear factor, erythroid 2; HOMX1: heme oxygenase 1.

The NRF2 signaling pathway is a critical defense mechanism against ferroptosis, contributing to ferroptosis resistance observed in multiple types of cancer cells [Figure 5]. Under normal conditions, NRF2 is retained in the cytoplasm by KEAP1, preventing its nuclear translocation and activation^[118]^. In certain types of cancer, mutations in KEAP1 can disrupt this interaction, resulting in elevated NRF2 activity^[118]^. Consequently, NRF2 may inhibit ferroptosis by enhancing the cell's antioxidant defenses^[119]^. Upregulation of dipeptidyl peptidase 9 (DPP9) increases the stabilization of NRF2, thus boosting sorafenib resistance in clear cell renal cell carcinoma cells^[120]^. Similarly, activation of Src tyrosine kinase stabilizes and activates NRF2, resulting in enhanced resistance of glioblastoma cells to ionizing radiation-induced ferroptosis^[121]^. In addition, NSUN2, an RNA m5C methyltransferase highly expressed in NSCLC, serves as an upstream of NRF2. Mechanistically, NSUN2 increases the m5C modification of NRF2 mRNA, leading to enhanced expression of NRF2^[122]^. Conversely, depleting NSUN2 decreases the expression of NRF2 and increases the sensitivity of NSCLC cells to ferroptosis activators both *in vitro* and *in vivo*^[122]^. Consistently, the NRF2 inhibitor trigonelline sensitizes chemoresistant head and neck cancer cells to ferroptosis inducers^[123]^.

Activation of NRF2 axis in cancer cells triggers the induction of multiple anti-ferroptotic genes, including SLC7A11, ATP binding cassette subfamily C member 5 (ABCC5), metallothionein 1G (MT1G), FSP1, CBS, and superoxide dismutase 2 (SOD2). In clear cell renal cell carcinoma cells, NRF2-mediated sorafenib resistance is largely dependent on its transcriptional target SLC7A11^[120]^. Similarly, NRF2 promotes the expression of multidrug resistance protein ABCC5 and metallothionein MT1G, leading to ferroptosis inhibition and sorafenib resistance in hepatocellular carcinoma cells^[124,125]^. NRF2 also promotes ferroptosis resistance by transcriptionally upregulating the expression of FSP1 in KEAP1-mutant lung cancers^[107,108]^. In erastin-resistant ovarian cancer cells, NRF2 transcriptionally upregulates CBS, the key enzyme in transsulfuration-mediated cysteine biosynthesis pathway^[126]^. Furthermore, cytochrome b-245 beta chain (CYBB), a subunit of NADPH oxidase, interacts with NRF2 and promotes temozolomide resistance by regulating the NRF2-SOD2 axis in mesenchymal glioblastoma^[127]^. Compensatory antioxidant SOD2 further protects against cytotoxicity induced by high ROS levels during ferroptosis in these temozolomide-resistant cancer cells^[127]^. In contrast, activating transcription factor 3 (ATF3) sensitizes gastric carcinoma cells to cisplatin via obstructing NRF2-SLC7A11 signaling^[128]^. These findings support that targeting the NRF2 axis offers a feasible strategy to overcome ferroptosis resistance in cancer therapy.

### Alteration in lipid metabolism

**Figure 6:**
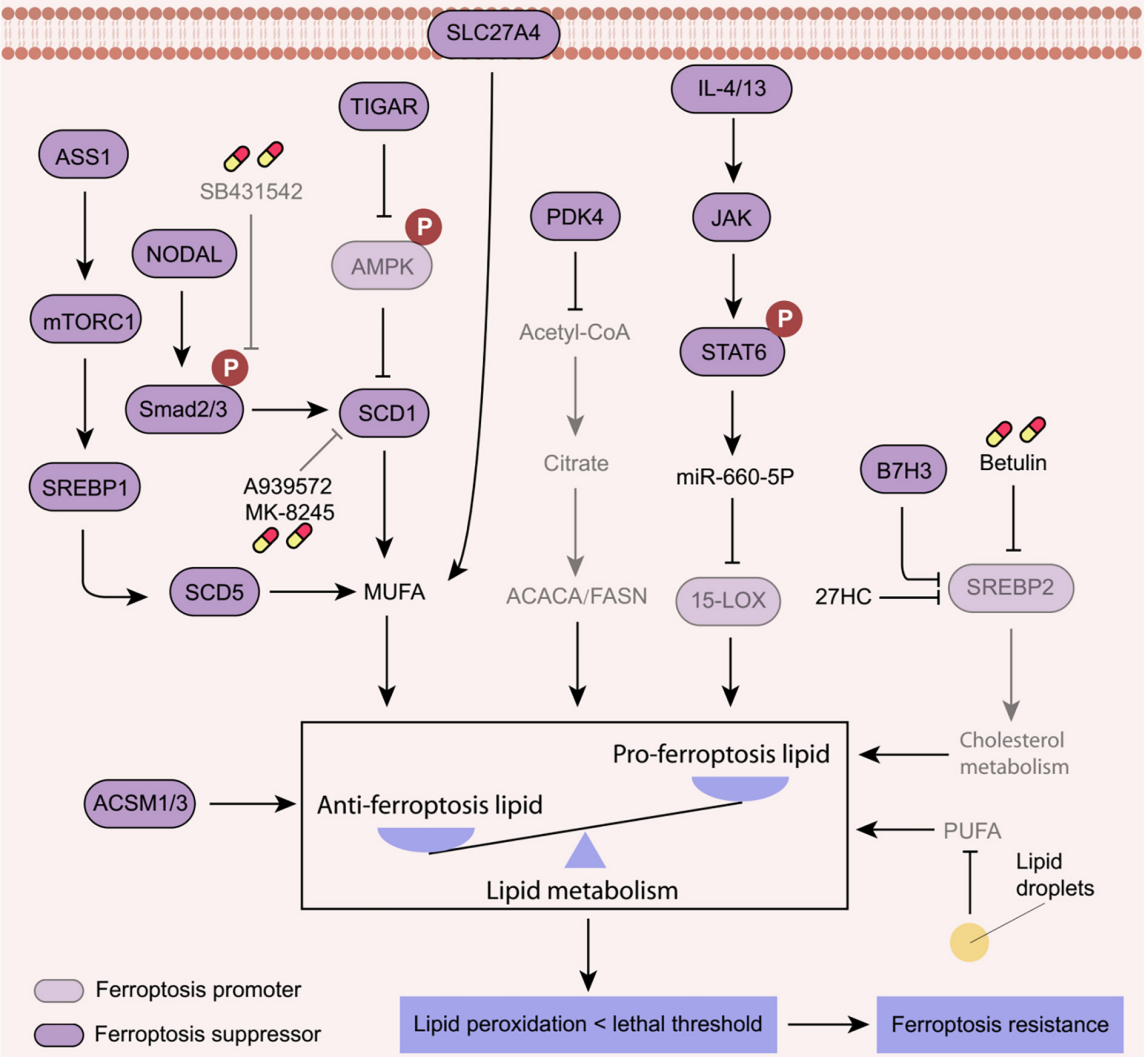
Ferroptosis resistance induced by alterations in lipid metabolism. PUFA is required for lipid peroxidation and ferroptosis, while the uptake and synthesis of MUFA contribute to ferroptosis resistance. Other pathways like lipid synthesis, cholesterol metabolism, and lipid droplet formation also determine the cancer cell sensitivity to ferroptosis. A939572 and MK-8245, SCD1 inhibitors; Betulin, a SREBP2 inhibitor; SB431542, a Smad2/3 inhibitor. PUFA: Polyunsaturated fatty acid; MUFA: monounsaturated fatty acid; SCD1: stearoyl-CoA desaturase 1; SLC7A11: solute carrier family 7 member 11; TIGAR: TP53-induced glycolysis regulatory phosphatase; ASS1: argininosuccinate synthase 1; SREBP1: sterol regulatory-element-binding protein 1; PDK4: pyruvate dehydrogenase kinase 4; ACSM1/3: acyl-CoA synthetase medium chain family member 1/3; SLC27A4: solute carrier family 27 member 4; IL4/13: interleukin 4/13; JAK: Janus kinase; STAT6: signal transducer and activator of transcription 6; 15-LOX: 15-lipoxygenase; PUFA: polyunsaturated fatty acid.

PUFA are structural components of cell membranes and act as substrates for lipid peroxidation. Alterations in lipid metabolism are closely associated with ferroptosis resistance [Figure 6]. Stearoyl-CoA desaturase 1 (SCD1), a multifunctional enzyme involved in lipid metabolism, inhibits ferroptosis by catalyzing the desaturation of unsaturated fatty acids to increase the levels of monounsaturated fatty acid (MUFA)^[129-131]^. TP53-induced glycolysis regulatory phosphatase (TIGAR), a p53 target gene, drives ferroptosis resistance in colorectal cancer by activating the ROS- AMP-activated protein kinase (AMPK)-SCD1 axis^[132]^. Moreover, SCD1 can be transcriptionally upregulated through the activation of SMAD family member 2 or 3 (Smad2/3) triggered by the overexpression of nodal growth differentiation factor (NODAL)^[131]^. Consequently, pharmacologic or genetic inhibition of SCD1 abolishes the ferroptosis resistance in colorectal cancer and melanoma cells^[131,133]^. Another member of the SCD family, stearoyl-CoA desaturase 5 (SCD5), plays a crucial role in ferroptosis resistance. In NSCLC cells, argininosuccinate synthase 1 (ASS1) activates the mTORC1-sterol regulatory-element-binding protein 1 (SREBP1)-SCD5 axis, promoting *de novo* MUFA synthesis and ferroptosis resistance^[134]^. Similarly, solute carrier family 27 member 4 (SLC27A4)-mediated uptake of MUFA facilitates the resistance to ferroptosis triggered by sorafenib in hepatocellular carcinoma^[135]^. Thus, targeting MUFA metabolism could be therapeutically beneficial in modulating ferroptosis resistance in cancer cells.

Intracellular lipid synthesis and storage are tightly regulated processes essential for normal cellular function. A screening using RNA interference library by targeting metabolic enzymes identified pyruvate dehydrogenase kinase 4 (PDK4) as an important gene involved in ferroptosis resistance^[136]^. PDK4 inhibits ferroptotic cell death by hindering pyruvate oxidation and fatty acid synthesis in human pancreatic ductal adenocarcinoma cells^[136]^. In prostate cancer, acyl-CoA synthetase medium chain family member 1 and 3 (ACSM1 and ACSM3) regulate lipidome and promotes resistance to ferroptosis through fatty acid oxidation^[137]^. Furthermore, cell cycle arrest has an inhibitory effect on ferroptosis in cancers by promoting lipid droplet formation, which sequesters excess PUFA to limit lipid peroxidation^[138]^. In contrast, lipophagy, the autophagy-mediated degradation of lipid droplets, facilitates ferroptotic cell death in cancer cells^[139]^. It may be very interesting to examine the crosstalk between cell cycle arrest and lipophagy in the ferroptosis process.

Hypercholesterolemia and disorders of lipid metabolism are associated with ferroptosis resistance in several cancers. Chronic exposure to 27-hydroxycholesterol (27HC), a metabolite of cholesterol, promotes ferroptosis resistance in breast cancer cells, leading to enhanced tumorigenesis and metastasis^[140]^. Additionally, B7H3/CD276, an immune checkpoint molecule, has been identified as a potential regulator of ferroptosis resistance in colorectal cancer cells^[141]^. B7H3 promotes ferroptosis resistance by modulating sterol regulatory element binding protein 2 (SREBP2)-mediated cholesterol metabolism^[141]^. However, this effect can be counteracted by exogenous cholesterol supplementation or the use of the SREBP2 inhibitor, betulin^[141]^. These findings emphasize the importance of cholesterol metabolism in controlling ferroptosis in cancer cells, highlighting its potential as a therapeutic target for ferroptosis resistance.

In the tumor microenvironment, lipid peroxidation in macrophages impairs their phagocytic ability to eradicate ferroptotic cancer cells, thereby promoting cancer resistance to ferroptosis^[142]^. Moreover, macrophage-derived exosomes can attenuate the expression of 15-LOX to limit ferroptosis in cervical cancer cells^[143]^. Additionally, KRAS^G12D^-containing exosomes released from ferroptotic pancreatic cancer cells cause macrophages to switch toward a pro-tumorigenesis M2-like phenotype by activating STAT3-dependent fatty acid oxidation^[144]^. Although the function of ferroptosis in regulating tumor immunity requires further elucidation, continued studies on lipid metabolism regulated-tumor microenvironment pathways will help to find new ways to overcome ferroptosis resistance.

### Alteration in iron metabolism

**Figure 7:**
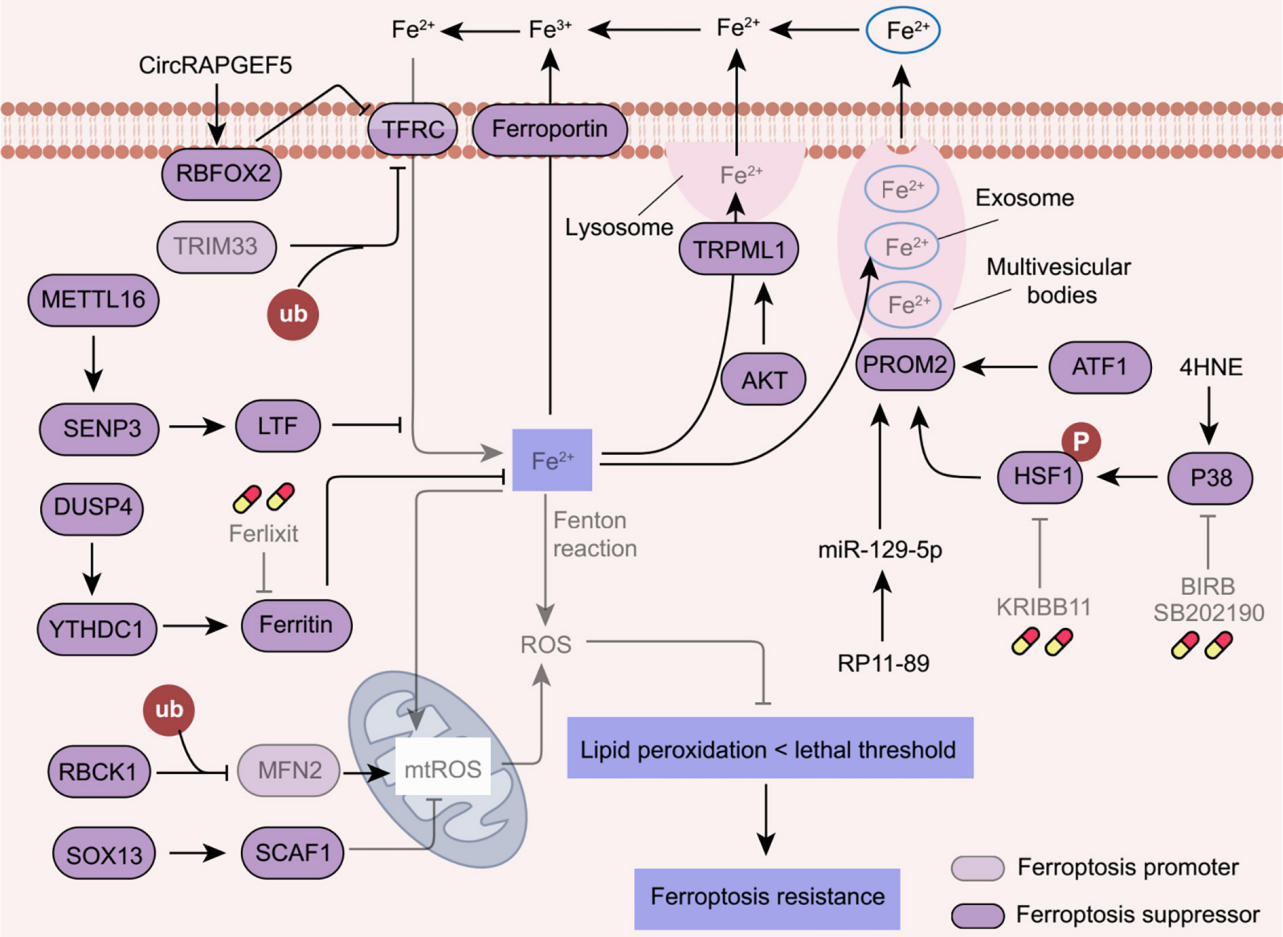
Ferroptosis resistance induced by alterations in iron metabolism. The upregulation of ferroportin, TRPML1, PROM2, and ferritin, or downregulation of TFRC, decreases intracellular free iron levels, leading to ferroptosis resistance in cancer cells. In addition, activation of RBCK1 and SOX13 may promote ferroptosis resistance by decreasing iron-mediated mitochondrial ROS. Ferlixit, an activator of ferritinophagy; BIRB and SB202190, P38 inhibitors; KRIBB11, a HSF1 inhibitor. TRPML1: Transient receptor potential mucolipin 1; PROM2: prominin 2; RBCK1: RANBP2-type and C3HC4-type zinc finger containing 1; SOX13: SRY-box transcription factor 13; HSF1: heat shock transcription factor 1; ATF1: activating transcription factor 1; TFRC: transferrin receptor; RBFOX2: RNA binding fox-1 homolog 2; METTL16: methyltransferase methyltransferase-like protein 16; SENP3: SUMO specific peptidase 3; DUSP4: dual-specificity phosphatase 4; YTHDC1: YTH N6-methyladenosine RNA binding protein C1; LTF: lactotransferrin; MFN2: mitofusin 2; SCAF1: SR-related CTD associated factor 1.

Although iron is crucial for various physiological processes, excess iron results in ROS production and lipid peroxidation, which promotes ferroptosis. Tumor cells often develop resistance to ferroptosis by manipulating iron metabolism [Figure 7].

Prominin 2 (PROM2) is a protein responsible for iron transport out of cells via multivesicular bodies (MVBs), thus inhibiting ferroptosis^[145]^. Overexpression of PROM2 increases metastatic potential and ferroptosis resistance in multiple cancers^[146]^. Strategies aimed at blocking PROM2 expression or function may enhance cancer cell sensitivity to ferroptosis induced by GPX4 inhibitors^[146]^. In lung cancer, activating transcription factor 1 (ATF1) promotes ferroptosis resistance by stabilizing PROM2 mRNA^[147]^. Additionally, the lipid peroxidation product 4-hydroxynonenal (4-HNE) enhances the expression of PROM2 by activating heat shock transcription factor 1 (HSF1)-dependent transcription^[148]^. Accordingly, HSF1 inhibitors have been shown to restore the sensitivity of breast cancer cells to ferroptosis^[148]^. Furthermore, LncRNA RP11-89/miR-129-5P inhibits ferroptosis via PROM2-activated iron export in bladder cancer^[149]^.

The iron transporter protein, TFRC, is considered the most important gene for intracellular iron uptake. CircRAPGEF5 binds the RNA-binding protein RNA binding fox-1 homolog 2 (RBFOX2), blocking RBFOX2-mediated splicing of TFRC pre-mRNA^[150]^. Therefore, elevated circRAPGEF5 levels decrease iron uptake and lipid peroxidation in endometrial cancer, leading to ferroptosis resistance^[150]^. Furthermore, the downregulation of the E3 ubiquitin ligase tripartite motif-containing 33 (TRIM33) leads to the stabilization of TFRC through reduced ubi quitination, thereby inhibiting ferroptosis in hepatocellular carcinoma^[151]^. Another study shows that the m6A methyltransferase methyltransferase-like protein 16 (METTL16) confers ferroptosis resistance in hepatocellular carcinoma^[152]^. Mechanistically, METTL16 catalyzes m6A modification of SUMO specific peptidase 3 (SENP3) mRNA, leading to enhanced stabilization of lactotransferrin (LTF), which subsequently reduces free iron^[152]^.

Ferritin, composed of the subunits ferritin heavy chain (FTH) and ferritin light chain (FTL), modulates iron metabolism by storing iron. FTH accelerates the growth and migration of hepatocellular carcinoma by conferring resistance to ferroptosis^[153]^. Mechanistically, FTH-reconstituted cells exhibit reduced lipid peroxidation, decreased mitochondrial ROS levels, and enhanced mitochondrial respiration^[153]^. Similarly, SRY-box transcription factor 13 (SOX13) and RANBP2-type and C3HC4-type zinc finger containing 1 (RBCK1) enhance resistance to ferroptosis in gastric and pancreatic cancers by boosting mitochondrial respiration^[154,155]^. Dual-specificity phosphatase 4 (DUSP4) inhibits sorafenib-induced ferroptosis in hepatocellular carcinoma by activating the RNA m6A reader, YTH N6-methyladenosine RNA binding protein C1 (YTHDC1), leading to enhanced expression of FTH and FTL^[156]^. In addition, extracellular matrix-detached conditions can facilitate ferroptosis resistance in cancer cells by activating the NRF2-FTH signaling^[157]^. In contrast, ferlixit, an iron compound, sensitizes ovarian cancer cells to ferroptosis by increasing ferritinophagy^[158]^. In addition, lysosome facilitates ferroptosis by generating free iron ions through the degradation of ferritin^[20,21]^. However, transient receptor potential mucolipin 1 (TRPML1) triggers lysosomal exocytosis, which reduces intracellular Fe^2+^ and promotes ferroptosis resistance in AKT-hyperactivated cancer^[159]^. Further investigation into the crosstalk between autophagy and ferroptosis could unveil novel therapeutic strategies to overcome ferroptosis resistance in cancer.

## CONCLUSION AND FUTURE PROSPECTS

Targeting ferroptosis pathways in tumor cells is emerging as a promising anticancer strategy. Despite extensive research into the mechanism underlying ferroptosis resistance in cancer cells, many questions remain unanswered. For instance, can ferroptosis inducers, as novel agents of cell death, be effective candidates in tumor therapy? How should we prioritize and target key molecules in ferroptosis pathways? Moreover, how can we precisely target the regulatory modulators that involve the ferroptosis-suppressing axis to overcome tumor resistance?

The practical implications of ferroptosis in the context of tumor resistance herald a new dawn in therapies targeting its key regulators. However, specific ferroptosis inhibitors have not yet entered clinical practice, and much of the research on ferroptosis resistance remains confined to preclinical studies. Several factors may account for this dilemma. Firstly, mitigating the toxicity and off-target effects of ferroptosis inducers continues to be a challenge. Secondly, the limited bioavailability and specificity of certain ferroptosis inducers impede their translation into clinical practice. Thirdly, tumor heterogeneity and variations in gene expression present substantial obstacles to the efficacy of targeted compounds across different cancer types, thereby diminishing their clinical applicability. Consequently, more extensive screening for potential ferroptosis inhibitors is crucial for advancing tumor therapy.

In conclusion, better insight into how tumor cells adapt and develop drug resistance will maximize the efficacy of pro-ferroptosis therapies.

## DECLARATIONS

### Copyright

© The Author(s) 2024.
